# A Case of Pulmonary Metastasis from Adenoid Cystic Carcinoma of the Hard Palate Exhibiting Ground Glass Nodules

**DOI:** 10.70352/scrj.cr.25-0203

**Published:** 2025-10-07

**Authors:** Kanjiro Murakami, Hiroshi Matsui, Kaori Ishida, Yuri Noda, Takahiro Utsumi, Natsumi Maru, Yohei Taniguchi, Tomohito Saito, Haruaki Hino, Koji Tsuta, Tomohiro Murakawa

**Affiliations:** 1 Department of Radiology, Kobe University Graduate School of Medicine, Kobe, Hyogo, Japan; 2 Department of Thoracic Surgery, Kansai Medical University Hospital, Hirakata, Osaka, Japan; 3 Department of Pathology and Laboratory Medicine, Kansai Medical University Hospital, Hirakata, Osaka, Japan; 4 Department of Thoracic Surgery, University of Tokyo Hospital, Tokyo, Japan

**Keywords:** ground-glass nodules, metastatic lung tumors, adenoid cystic carcinoma

## Abstract

**INTRODUCTION:**

Metastatic lung tumors typically exhibit well-defined, enhanced margins on chest CT. They may rarely present as ground-glass nodules (GGNs), making it challenging to differentiate from primary lung cancers, which can also manifest as progressively enlarging GGNs on CT.

**CASE PRESENTATION:**

The patient is a woman in her 50s who underwent surgery for adenoid cystic carcinoma of the hard palate 19 years ago. Seven years ago, chest CT showed GGNs in the upper lobe of the right lung as well as in the upper and lower lobes of the left lung. The patient underwent bilateral lung wedge resection for multiple lung nodules. The pathological diagnosis of the lung nodules was metastases from adenoid cystic carcinoma of the hard palate. In addition, chest CT performed 3 years ago revealed solid nodules in the left lung (S1+2/10), and a follow-up CT performed 1 year ago showed a solid nodule in the right lung (S1). Consequently, partial lung resections were performed for each lesion via thoracoscopic surgery. The pathological results indicated a metastatic adenoid cystic carcinoma. Recently, an enlarging GGN was found in the left lung (S4), which raised suspicion of primary lung cancer. Therefore, thoracoscopic partial resection of the left lingular segment was performed. The final pathology confirmed pulmonary metastasis of adenoid cystic carcinoma.

**CONCLUSIONS:**

In cases where a patient has a history of malignancy and a GGN on chest CT, metastatic lung tumors should be included in the differential diagnosis.

## Abbreviation

GGN
: ground glass nodule

## INTRODUCTION

Generally, metastatic lung tumors appear as multiple solid nodules with well-defined margins on chest CT. Primary lung cancer is the most common differential diagnosis for chest CT showing GGNs, whereas metastatic lung tumors are rare. We herein report a case of lung metastasis from adenoid cystic carcinoma presenting with GGNs.

## CASE PRESENTATION

A woman in her 50s underwent resection and reconstruction for adenoid cystic carcinoma of the hard palate 19 years ago. Seven years ago, a chest X-ray performed during a medical check-up showed an abnormal shadow, and a chest CT revealed pure GGNs in the right (S1) and left (S3) lung, as well as a part-solid GGN in the left (S6) lung. She was referred to the Division of Respiratory Medicine, Infectious Disease, and Allergology for further evaluation of infectious diseases. However, a definitive diagnosis was not made, and she was referred to our department.

**Fig. 1:**
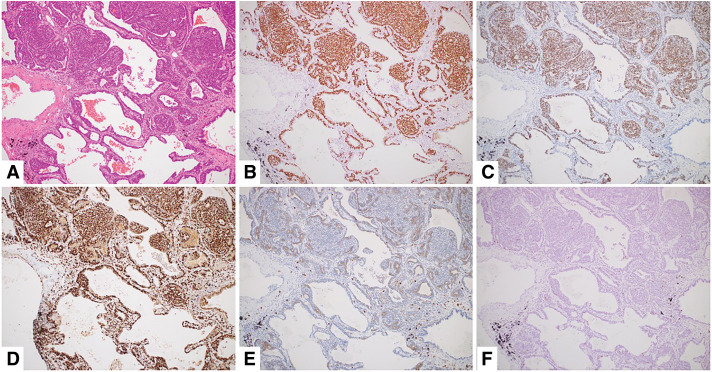
The tumor showing a cribriform pattern with pseudocystic spaces formed by the 2 cell types (hematoxylin and eosin staining, ×10) (**A**). An immunohistochemical examination showed that the tumor cells were positive for p63, SOX10, MYB, and CD117 (**B**–**E**). Ki-67 expression was observed in less than 10% of tumor cells (**F**), with no significant difference between the solid and ground-glass nodule components.

Because of suspicion of multiple lung adenocarcinomas due to GGNs, partial resection of the left lung (S3/6) and right lung (S1) was performed via a 2-stage surgery. The tumors consisted of biphasic proliferation of basaloid myoepithelial-like cells and ductal epithelial cells. The tumors showed peribronchial and interstitial infiltration, and a cribriform pattern with pseudocystic spaces formed by the 2 cell types was observed. The myoepithelial-like tumor cells were positive for p63, SOX10, and MYB. The ductal epithelial tumor cells were positive for CD117. Based on the above findings, the lung nodules were diagnosed as metastatic adenoid cystic carcinoma of the hard palate (**Fig. 1**).

Partial thoracoscopic resection was performed on the S1+2/10 of the left lung and on the S1 of the right lung 3 and 1 year ago, respectively. Preoperative chest CTs showed that the lung nodules were well-defined and solid, typical of metastatic lung tumors. The pathological results indicated pulmonary metastases of adenoid cystic carcinoma of the hard palate.

**Fig. 2:**
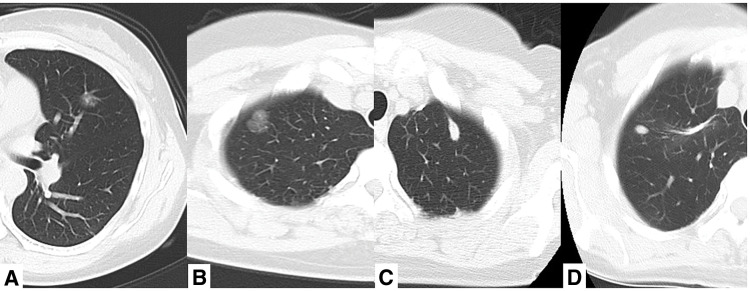
Pulmonary metastasis of adenoid cystic carcinoma. Chest CT showed GGNs or solid nodules. At present: 14-mm GGN in the S4 of the left lung (**A**); 7 years ago: 16-mm GGN in the S1 of the right lung (**B**); 3 years ago: 13-mm solid nodule in the S1+2 of the left lung (**C**); 1 year ago: 8-mm solid nodule in the S1 of the right lung (**D**). GGN, ground-glass nodule

At this time, chest CT showed an enlarging GGN in the lingual region (S4) of the left lung, which raised suspicion of primary lung cancer. However, the previous history suggested the possibility of metastasis from adenoid cystic carcinoma of the hard palate. Thus, the decision to perform partial thoracoscopic resection of the lingual region of the left lung was made (**Fig. 2**).

She showed no systemic symptoms, and her physical examination and laboratory results were normal. We performed wedge resection of the lingual region of the left lung via video-assisted thoracic surgery. Her postoperative course was uneventful, and she was discharged on POD 4. No cancer recurrence was observed over 20 months from the last lung resection.

**Fig. 3:**
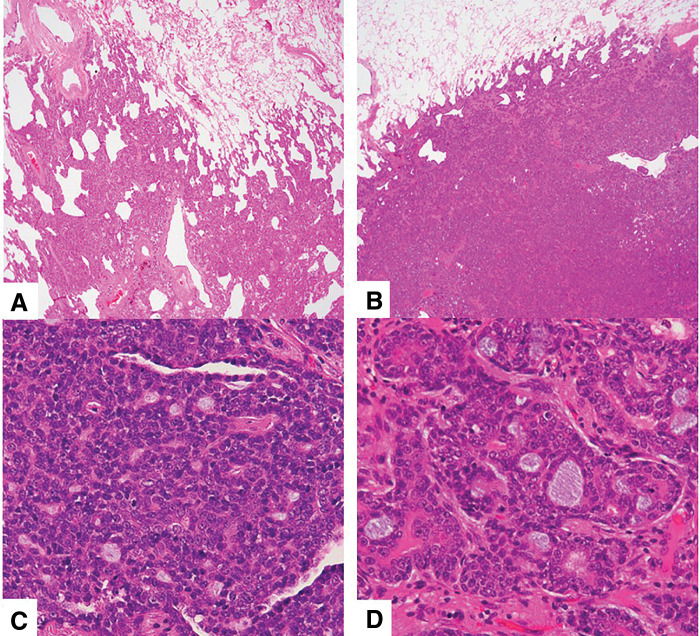
Tumor growth pattern. Lepidic growth at present and 7 years ago (hematoxylin and eosin staining, ×20 (**A**) and ×400× (**C**)). Solid growth 3 and 1 year ago (hematoxylin and eosin staining, ×20 (**B**) and ×400 (**D**)). The MIB-1 index was 3.4% in the lepidic component, and 1.8% in the solid component. MIB-1, mindbomb homolog-1

Macroscopically, the tumor was an elastic, hard, grayish-white nodule with a diameter of 10 mm and a lung resection margin of 25 mm. On the final histopathological examination, the central part of the tumor showed the formation of tumor nests with a mixture of true lumens lined by ductal epithelial cells against a background of eosinophilic stroma and pseudo-lumens lined by tumor myoepithelial cells. At the periphery, infiltration along the alveolar septa was observed, which led to the diagnosis of metastatic adenoid cystic carcinoma. Furthermore, the tumors resected 7 years ago showed a predominantly lepidic growth pattern with stromal tumor invasion, whereas those from 3 and 1 year ago presented a solid growth pattern (**Fig. 3**).

## DISCUSSION

Metastatic lung tumors typically present on CT as multiple peripheral and solid nodules. The present case is reported due to GGN on CT despite being a metastasis from a previous adenoid cystic carcinoma of the hard palate. GGN is defined as “a radiologic finding showing a hazy opacity with the presence of the underlying pulmonary vessels or bronchial structures,” and is pathologically explained by (1) partially inflated alveoli with thickened interstitium, (2) incomplete alveolar filling due to inflammatory cells or exudate, and (3) partial alveoli collapse.^1–3)^ The differential diagnoses for GGN include atypical adenomatous hyperplasia; adenocarcinoma *in situ* involving type 2 pneumocytes or Clara cells with replacement growth; minimally invasive adenocarcinoma; invasive adenocarcinoma, particularly the lepidic predominant type; primary lung adenocarcinoma with metastatic lung tumors; pneumonia; and diffuse alveolar hemorrhage.

Metastatic lung tumors presenting as GGN have been reported in gastric, pancreatic, breast, and renal cancers, as well as malignant melanoma. The factors contributing to the presentation of these metastatic lung tumors as GGN on CT include ① mucin-producing tumors in which loosely cohesive tumor cells containing abundant mucin irregularly and incompletely fill the alveoli; ② lymphatic spread leading to tumor emboli in the lymphatic vessels of the interlobular septa, thereby causing retrograde flow obstruction and interstitial edema; and ③ proliferation of tumor cells replacing the lung parenchymal epithelium.^4–8)^ In this case, the tumor cells were proliferating along the alveolar septa, similar to ③, leading to the appearance of GGN on chest CT.

Adenoid cystic carcinoma predominantly occurs among women and in individuals aged 50–60 years. It mainly arises in major salivary glands, such as the submandibular, sublingual, and minor salivary glands;^9)^ however, pulmonary origin is exceedingly rare. Preoperative diagnosis is possible through fine-needle aspiration cytology.^10)^ This carcinoma is pathologically characterized by myoepithelial cells with a mixed pattern of basaloid and glandular epithelial cells, exhibiting cribriform, tubular, and solid patterns.^9)^ It gradually metastasizes over 5 years postoperatively, with approximately 40%–70% of cases showing metastasis to the lungs.^11–13)^ In the present case, the primary site was the hard palate, and the patient underwent long-term follow-up in otolaryngology.

In our search of medical journals, lung metastases of adenoid cystic carcinoma showing GGN were observed in only 1 reported case, which originated from breast cancer.^14)^ There were no reports on adenoid cystic carcinoma lung metastases originating from head and neck primaries showing GGN.

In this case, images of metastatic lung tumors were presented, alternating between GGNs and solid nodules. Similar reports have been documented in pulmonary pleomorphic carcinoma, where differences in CT images were attributed to varying proportions of tumor cells.^15)^ Because of the variations in tumor histological growth patterns, different CT findings were observed, such as solid nodules and GGNs, in this case. However, the underlying reasons remain unclear. This was not attributed to differences in tumor malignancy, but rather to the inherent difference in their histological growth patterns, which directly impacts their CT appearance. Furthermore, there were no other discernible differences in the background lung pathology.

## CONCLUSIONS

We herein present a case of adenoid cystic carcinoma of the hard palate with lung metastases presenting as GGN on chest CT. The reasons for the alternating appearances of GGNs and solid nodules were not definitively explained. However, when lung nodules present as GGN on chest CT, particularly in a patient with a history of malignant tumors, the differential diagnosis of metastatic lung tumors should be considered.

## DECLARATIONS

### Availability of data and materials

All datasets presented in the main paper are available whenever possible.
